# Retraction

**DOI:** 10.1002/cam4.6367

**Published:** 2023-09-19

## Abstract

Zhang Z, Tan X, Luo J, Cui B, Lei S, Si Z, Shen L, Yao H. GNA13 promotes tumor growth and angiogenesis by upregulating CXC chemokines via the NF‐κB signaling pathway in colorectal cancer cells. Cancer Med. 2018;7(11):5611‐5620. https://onlinelibrary.wiley.com/doi/full/10.1002/cam4.1783. The above article, published online on September 28, 2018, in Wiley Online Library (wileyonlinelibrary.com), has been retracted by agreement between the authors, the journal's Editor‐in‐Chief, Dr Stephen Tait, and John Wiley & Sons Ltd. The retraction has been agreed due to evidence of image duplication between figures 3D, 3E, and 6C and figures from articles published elsewhere in a different scientific context. As a result, the journal no longer has confidence in the results and conclusions.

Zhang Z, Tan X, Luo J, Cui B, Lei S, Si Z, Shen L, Yao H. GNA13 promotes tumor growth and angiogenesis by upregulating CXC chemokines via the NF‐κB signaling pathway in colorectal cancer cells. Cancer Med. 2018;7(11):5611‐5620. https://onlinelibrary.wiley.com/doi/full/10.1002/cam4.1783. The above article, published online on September 28, 2018, in Wiley Online Library (wileyonlinelibrary.com), has been retracted by agreement between the authors, the journal's Editor‐in‐Chief, Dr Stephen Tait, and John Wiley & Sons Ltd. The retraction has been agreed due to evidence of image duplication between figures 3D, 3E, and 6C and figures from articles published elsewhere in a different scientific context. As a result, the journal no longer has confidence in the results and conclusions.

